# Automated macular choroidal thickness measurement by swept-source optical coherence tomography in pseudoxanthoma elasticum

**DOI:** 10.1186/s40942-016-0040-0

**Published:** 2016-06-13

**Authors:** Rosa Dolz-Marco, María Andreu-Fenoll, Pablo Hernández-Martínez, M. Dolores Pinazo-Durán, Roberto Gallego-Pinazo

**Affiliations:** 1 Unit of Macula, Department of Ophthalmology, University and Polytechnic Hospital La Fe, Bulevar Sur s/n, 46026 Valencia, Spain; 2 RETICS RD12/0034 Enfermedades oculares: «Prevención, detección precoz y tratamiento de la patología ocular prevalente degenerativa y crónica», Instituto de Salud Carlos III, Madrid, Spain; 3 Ophthalmology Research Unit “Santiago Grisolía”, University Hospital Doctor Peset, Valencia, Spain; 4 Department of Surgery, Faculty of Medicine, University of Valencia, Valencia, Spain

**Keywords:** Automated segmentation, Choroid, Neovascularization, PXE

## Abstract

**Introduction:**

Pseudoxanthoma elasticum (PXE) typically involves elastic fibers in blood vessels and Bruch membrane. Our purpose was to analyze retinal and choroidal macular thickness in patients with angioid streaks due PXE compared with a control group.

**Methods:**

Best-corrected visual acuity (BCVA), axial length (AL), and macular swept-source optical coherence tomography were obtained. Automated segmentations of the retina and the choroid were used to obtain the corresponding thickness values. An age, gender and AL matched control group was used to compare the thickness values.

**Results:**

Twelve eyes of 6 patients were included. The mean BCVA was 0.68 ± 0.29 versus 1.0 in controls (p < 0.001). The mean macular retinal thickness was thinner in eyes with PXE (p = 0.038). Only patients with choroidal neovascularization (NV) showed statistically significant differences in the mean macular choroidal thickness (p = 0.008).

**Conclusions:**

The present study shows that choroidal thickness may be thinner in eyes with NV due to angioid streaks in PXE compared with healthy eyes analyzed by an automated segmentation of the choroid. Further studies are warranted in order to assess the importance of this choroidal changes in the pathogenesis of retinal disturbances related to PXE and its influence in long-term follow-up.

## Background

Pseudoxanthoma elasticum (PXE) is a systemic disease characterized by progressive fragmentation and calcification of the elastic fibers in connective tissues related to a mutation in the ABCC6 gene, resulting in a spectrum of pathologic changes mainly involving the dermis, the blood vessels, and the Bruch membrane [1, 2].

The eye is a typical target of PXE, with classic ocular signs such as *peau d’orange* degeneration, areas of chorioretinal atrophy, choroidal neovascularization, and angioid streaks [2–4]. The advent of multimodal imaging of the fundus led to the description of further signs that can be observed with fundus autofluorescence (FAF)—pattern dystrophy of the retinal pigment epithelium (RPE), pigmentary changes within the angioid streaks [5], or optical coherence tomography (OCT)—diffuse vitelliform deposits, intraretinal pigment migration, pockets of non-exudative subretinal fluid [5, 6]. Also, multimodal imaging of the fundus in patients with PXE enable a more objective and precise classification and identification of all these findings and their severity, thus making possible to establish an accurate prognosis and therapeutic indication when needed.

The choroid has one of the highest flows in the body [7]. Although Bruch membrane is thought to be particularly involved in PXE, given the systemic vascular changes related to PXE, it is rationale to believe that the choroid might somehow be involved [8]. With the swept-source (SS) laser technology in OCT, high-speed scanning rate and low-sensitivity roll-off versus depth compared to conventional spectral-domain technology has been introduced [9]. Thus, SS-OCT obtains a high-contrast image of the entire choroid making possible a precise delineation of the sclero-choroidal boundary [9, 10]. In the present study the retinal and choroidal thickness of the macular region in eyes with angioid streaks secondary to PXE were analyzed and compared with control eyes. We also evaluated the influence of the choroidal changes in cases showing NV. The use of an automated segmentation of the choroid provides an easier and accurate analysis of the choroidal thickness compared with previous reports.

## Methods

The present study was conducted in accordance with the principles of the Declaration of Helsinki and in compliance with the local institutional review board of the Institute of Health Research at the University and Polytechnic Hospital La Fe. Informed consent was obtained from all patients.

Patients diagnosed with angioid streaks due to PXE were included in the present observational retrospective cross-sectional study performed at the University and Polytechnic Hospital La Fe. The diagnosis of PXE was previously confirmed by skin biopsy and genetic test. An age and gender matched group was used as a control group, excluding patients with history or evidence of any primary or secondary ocular disease, or history of any intraocular surgery.

All patients underwent a comprehensive ophthalmic examination including best-corrected visual acuity (BCVA) using standard early treatment of diabetic retinopathy study (ETDRS) charts (decimal scale), axial length (AL) measurement (IOLMaster 500; Carl Zeiss Meditec, Inc, Dublin, CA), and color fundus and autofluorescence photography (Visupac, Carl Zeiss Meditec Inc, Dublin, CA). All patients were scheduled for a tomographic analysis of the whole macular region with the SS-OCT (DRI OCT-1 Atlantis; Topcon, Tokyo, Japan) at 1050 nm wavelength.

**Fig. 1:**
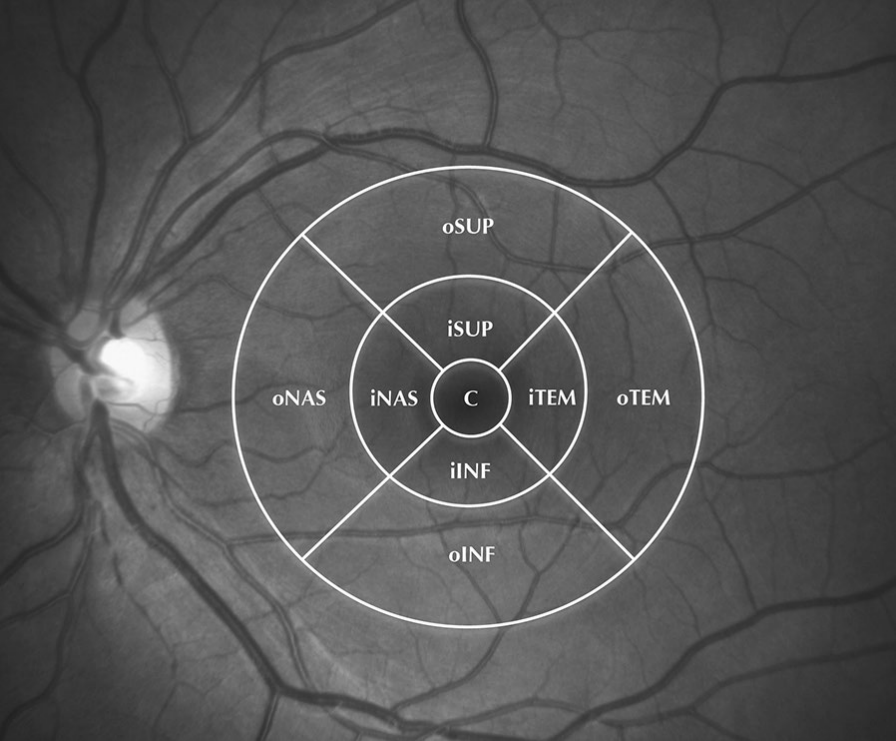
In the present study a modified ETDRS grid (6 × 6 mm) was used overlapped to the macular retinal and choroidal thickness maps. The grid was subdivided in nine sectors: a central sector (*C*) within the 500 μm from the fovea; 4 inner or juxtafoveal sectors within 500–1500 μm from the fovea (*iSUP*, *iTEM*, *iINF*, *iNAS*); and 4 outer or extrafoveal sectors within 1500–3000 μm from the fovea (*oSUP*, *oTEM*, *oINF*, *oNAS*)

Trained examiners performed the 3D raster scan modality with SS-OCT after pupil dilation. This scan protocol produces a retinal and choroidal tomography map with 512 horizontal and 256 vertical A-scans. This allows obtaining a retinal and choroidal thickness map of the entire macular area (12 × 9 mm) after an automated segmentation of the retinal layers including the choroid. The SS-OCT scans were analyzed in order to verify that the automated delineation had located the reference properly in the inner and outer boundary of the retina and the choroid. The retinal and choroidal thickness maps were overlapped to an ETDRS grid (6 × 6 mm) in order to study the different mean values of each sector (Fig. 1). The mean thickness of each sector was automatically measured within 500 microns (μm) from the center of the fovea in the central sector; 500–1500 μm from the center of the fovea in the 4 juxtafoveal sectors (nasal, inferior, temporal, and superior), and 1500–3000 μm from the center of the fovea in the 4 extrafoveal sectors (nasal, inferior, temporal, and superior).

Data were processed and the patients with PXE were subdivided into three different groups regarding the presence of neovascularization (NV) confirmed by fluorescein angiography and SS-OCT (Group 1), the presence of subretinal fluid (SRF) in the absence of NV confirmed by fluorescein angiography and SS-OCT (Group 2) or the absence of both NV and SRF (Group 3).

All data were collected in an Excel document (Microsoft^®^ Excel^®^ 2016 for Mac, version 15.14). Statistical analysis was performed using the using IBM^®^ SPSS^®^ statistics software for Mac (Version 20.0.0). Non parametric Wilconxon test was used to compared the mean retinal and choroidal thickness between PXE patients and the control group. The intergroup analysis was performed using Non parametric Kruskal–Wallis test. A p value ≤0.05 was consider statistically significant.

## Results

Twelve eyes of 6 patients diagnosed with angioid streaks related to PXE (3 men and 3 women) with a mean age of 40.66 ± 16.25 years were included in the present study. As a control group, 30 eyes of 15 patients (7 men and 8 women) with a mean age of 41.47 ± 12.88 years (p = 0.539) were evaluated. The mean BCVA was 0.68 ± 0.29 in eyes with PXE and 1.0 in all the patients of the control group (p < 0.001). The mean AL was 23.57 ± 0.91 mm in eyes with PXE and 23.36 ± 0.93 mm in the control group (p = 0.474). Four eyes (33.3 %) showed the presence of NV (Group 1); 2 eyes (16.7 %) evidenced the presence of SRF (Group 2); and 6 patients (50 %) showed no NV or SRF (Group 3).

The mean automatically measured macular retinal thickness was 269.03 ± 17.03 μm in eyes with PXE and 281.99 ± 12.27 μm in the control group (p = 0.038). The mean automatically measured retinal thickness of the central sector of the ETDRS grid (within 500 μm from the center of the fovea) was 231.42 ± 48.44 μm in eyes with PXE and 238.73 ± 16.12 μm in the control group (p = 0.707). The mean retinal thickness of the nasal, inferior, temporal and superior sectors, automatically measured in the juxtafoveal area (500–1500 μm from the center of the fovea), and in the extrafoveal area (1500–3000 μm from the center of the fovea) are summarized in the Table 1.

**Table 1:** Mean retinal and choroidal thickness values per sector

Sector	Study eye	Control group	p value
	n = (12)	n = (30)	
*Retina*			
Central	231.42 ± 48.44	238.73 ± 16.12	0.707
Inner nasal	293.70 ± 18.31	309.67 ± 13.11	0.018
Inner inferior	270.60 ± 22.05	285.41 ± 13.70	0.048
Inner temporal	288.60 ± 28.38	306.53 ± 13.20	0.111
Inner superior	247.30 ± 12.18	260.20 ± 16.87	0.009
Outer nasal	276.90 ± 25.70	296.97 ± 12.77	0.007
Outer inferior	240.60 ± 14.29	259.07 ± 18.22	0.002
Outer temporal	292.20 ± 17.36	309.27 ± 15.92	0.006
Outer superior	253.10 ± 13.72	272.97 ± 17.19	0.001
Mean	269.03 ± 17.03	281.99 ± 12.27	0.038
*Choroid*			
Central	225.50 ± 76.96	325.63 ± 86.91	0.003
Inner nasal	188.10 ± 83.57	304.20 ± 88.14	0.002
Inner inferior	133.60 ± 67.13	258.00 ± 85.26	<0.001
Inner temporal	232.50 ± 92.56	318.53 ± 81.89	0.011
Inner superior	219.70 ± 98.20	313.43 ± 82.74	0.005
Outer nasal	245.50 ± 80.91	315.17 ± 83.23	0.065
Outer inferior	234.40 ± 64.05	296.17 ± 72.32	0.031
Outer temporal	238.80 ± 74.22	314.73 ± 91.86	0.018
Outer superior	255.30 ± 82.32	323.13 ± 76.62	0.03
Mean	217.27 ± 71.86	307.27 ± 74.19	0.002

Comparison between eyes with angioid streaks and the control group

The mean automatically measured macular choroidal thickness was 217.27 ± 71.86 μm in eyes with PXE and 307.26 ± 74.19 μm in the control group (p = 0.002). The mean automatically measured choroidal thickness of the central sector of the ETDRS grid (within 500 μm from the center of the fovea) was 225.50 ± 76.96 μm in eyes with PXE and 325.63 ± 86.91 μm in the control group (p = 0.001). The mean choroidal thickness of the nasal, inferior, temporal and superior sectors, automatically measured in the juxtafoveal area (500–1500 μm from the center of the fovea), and in the extrafoveal area (1500–3000 μm from the center of the fovea) are summarized in the Table 1.

**Fig. 2:**
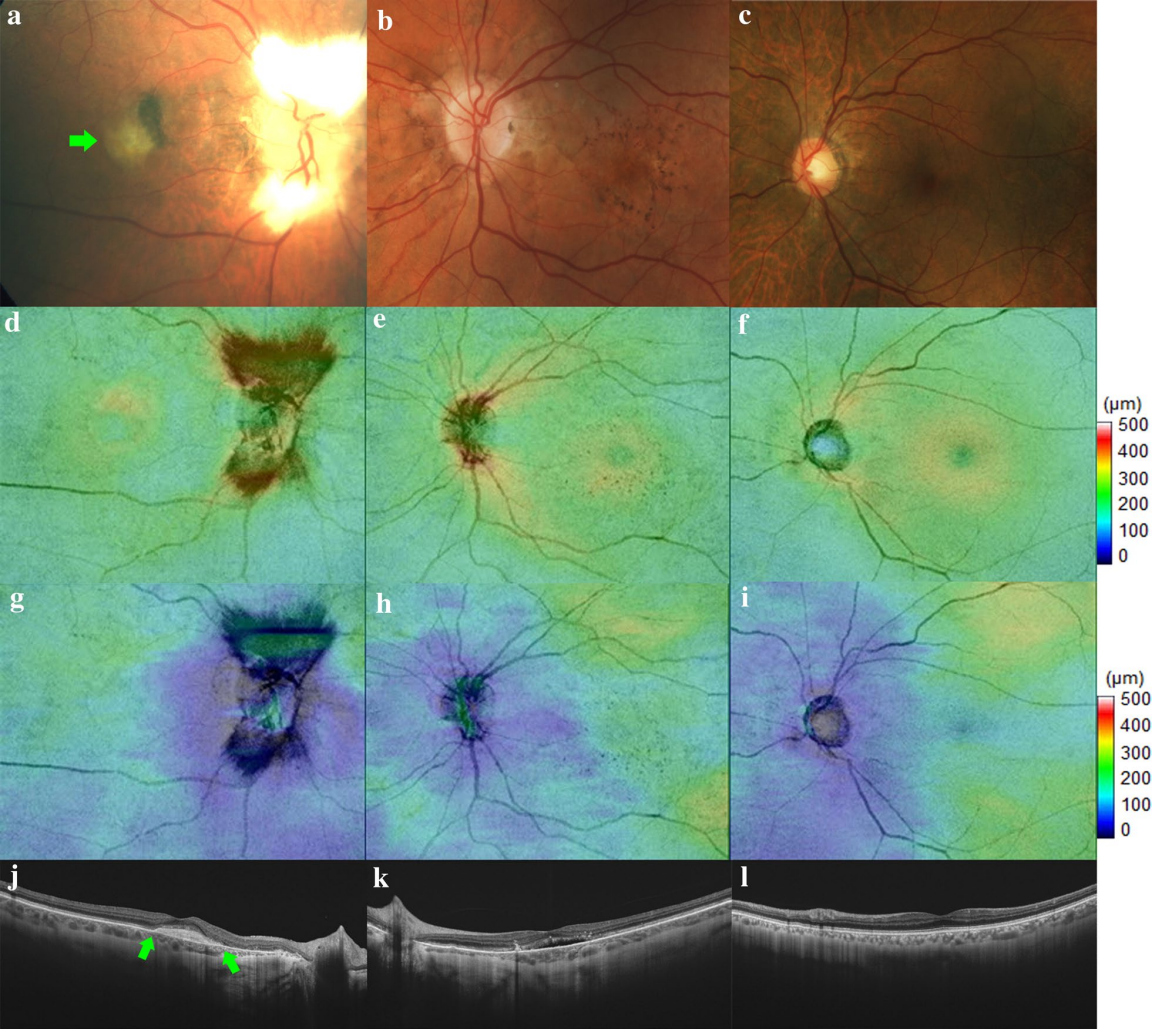
The *first color* fundus photography (**a**) demonstrates the presence of a subfoveal choroidal neovascularization in a patient with angioid streaks due to Pseudoxantoma elasticum (Group 1). The second case (**b**) shows pigmentary changes associated to angioid streaks (Group 2). The third case (**c**) evidences the presence of angioid streaks without other association (Group 3). The retinal and choroidal thickness maps (**d**–**i**) evidence in all cases a normal topographic distribution. The swept-source OCT horizontal scan centered in the fovea in the first case (**j**) shows the presence of a subretinal fibrosis with no signs of exudative activity; the second scan (**k**) shows the presence of subretinal fluid associated with pigment migration; the third scan (**l**) shows a normal macular anatomy and the absence of NV or SRF

The intergroup analysis (comparison between patients with/without NV o SRF) did not show any significant differences in the mean macular and central automatically measured retinal or choroidal thickness (Fig. 2). These data are summarized in Table 2. In addition, in the comparison of each subgroup with the control group, only Group 1 (those patients with NV) has shown statistically significant differences in the mean automatically measured macular choroidal thickness (p = 0.008) and in the mean automatically measured choroidal thickness of the central sector of the ETDRS grid (p = 0.01).

**Table 2:** Intergroup analysis of the retinal and choroidal thickness measured in the central sector of the ETDRS grid (within 500 μm from the center of the fovea) and the mean macular retinal and choroidal thickness

Sector	Group 1	p value*	Group 2	p value*	Group 3	p value*	Control group	p value^≈^	p value	p value^§^
	n = (4)		n = (2)		n = (6)		n = (30)			
*Retina*										
Central	201.00 ± 63.59	0.180	286,50 ± 24.75	0.030	233,33 ± 25.59	0.480	238.73 ± 16.12	0.064	0.088	0.517
Mean	264.61 ± 21.15	0.087	273.33 ± 16.18	0.459	270.54 ± 17.08	0.161	281.99 ± 12.27	0.360	0.990	0.520
*Choroid*										
Central	184.75 ± 30.51	0.010	228.50 ± 51.62	0.120	251.67 ± 98.92	0.120	325.63 ± 86.91	0.165	0.990	0.394
Mean	184.42 ± 31.34	0.008	210.11 ± 52.48	0.102	241.56 ± 92.89	0.090	307.27 ± 74.19	0.360	0.990	0.290

* p value: comparison between each group and the control group

^≈^p value: comparison between Group 1 and Group 2

^¶^p value: comparison between Group 2 and Group 3

^§^p value: comparison between Group 1 and Group 3

## Discussion

The choroid is an essential tissue for the proper function of the retina. The classical diagnostic methods including fluorescein and indocyanine green angiographies, spectral domain OCT and ultrasonography provide incomplete information regarding the anatomy and function of the choroid [11]. However, the advent of new OCT devices such as enhanced depth imaging and SS-OCT has led to improve the visualization and knowledge of the choroidal anatomy in different retinal diseases [8].

The role of the choroid in the pathogenesis of the ocular involvement secondary to angioid streaks due to PXE is not well understood. Ellabban et al. [8] analyzed the manually measured choroidal thickness in cases of angiod streaks due to PXE and reported the presence of a thinner choroid in cases of NV associated with PXE. Other authors analyzed the changes on choroidal thickness in patients with PXE compared based on a classification of the grade of Bruch’s membrane damage, concluding that the severity of the Bruch’s membrane calcification was associated with thinner choroidal thickness [12, 13]. Our results are consistent with these findings, showing a significant thinning in cases with NV. An automatically measurement of the choroidal thickness by SS-OCT software was performed in the present study compared with previous studies analyzing the choroidal thickness with manual measuring. We found a significant decrease in the mean macular choroidal thickness and in the mean choroidal thickness measured in the central sector of the ETDRS grid in patients with NV due to PXE. Those cases with SRF or in absence of NV or SRF did not show significant differences in the choroidal thickness values compared with the control group, although the mean thickness values were lower in patients with PXE. We hypothesized that the development of NV may be related to a greater impairment of the choroidal tissue, however the direct relationship of choroidal thinning and the development of NV is still not well established. Also the analysis of qualitative changes in the choroid should be assessed over a long-term follow-up in order to evaluate the presence of dilated great choroidal vessels even in the presence of thin choroids as seen in other pathologies in the spectrum of pachychoroid diseases [14].

The main limitation of the current study is the small number of patients and the lack of follow up due to the design of the study. However, our results obtained by an automatically measurement of the retinal and choroidal thickness in patients with PXE are consistent with previous reports using manual measurement of the choroid. The manual assessment of the choroidal thickness may minimize the errors related to automatic procedures, however is time consuming and may not be possible outside research projects. With the automatic measurement of the choroidal thickness provided by the SS-OCT we were able to analyze a 3D map of the entire macular area.

## Conclusions

The automatic segmentation of the choroidal tissue in cases with PXE may provide an accurate and clinically valuable information without further analysis and processing of the images. Further studies with a larger number of patients are warranted. Also the analysis of potential changes in the choroidal thickness in patients with PXE over time should be performed in order to better understand the pathogenesis of NV in patients with angioid streaks due to PXE and its potential relationship with the grade of damage of the Bruch’s membrane.

## Abbreviations

AL: axial length
BCVA: best-corrected visual acuity
ETDRS: early treatment of diabetic retinopathy study
FAF: fundus autofluorescence
NV: neovascularization
OCT: optical coherence tomography
PXE: pseudoxanthoma elasticum
SRF: subretinal fluid
SS: swept-source

## References

[1] Le Saux O, Urban Z, Tschuch C, Csiszar K, Bacchelli B, Quaglino D (2000). Mutations in a gene encoding an ABC transporter cause pseudoxanthoma elasticum. Nat Genet.

[2] Finger RP, Charbel Issa P, Ladewig MS, Götting C, Szliska C, Scholl HP (2009). Pseudoxanthoma elasticum: genetics, clinical manifestations and therapeutic approaches. Surv Ophthalmol.

[3] Clarkson JG, Altman RD (1982). Angioid streaks. Surv Ophthalmol.

[4] Dreyer R, Green WR (1978). The pathology of angioid streaks: a study of twenty-one cases. Trans Pa Acad Ophthalmol Otolaryngol.

[5] Charbel Issa P, Finger RP, Holz FG, Scholl HP (2009). Multimodal imaging including spectral domain OCT and confocal near infrared reflectance for characterization of outer retinal pathology in pseudoxanthoma elasticum. Invest Ophthalmol Vis Sci.

[6] Zweifel SA, Imamura Y, Freund KB, Spaide RF (2011). Multimodal fundus imaging of pseudoxanthoma elasticum. Retina.

[7] Linsenmeier RA, Padnick-Silver L (2000). Metabolic dependence of photoreceptors on the choroid in the normal and detached retina. Invest Ophthalmol Vis Sci.

[8] Ellabban AA, Tsujikawa A, Matsumoto A, Ogino K, Hangai M, Ooto S (2012). Macular choroidal thickness and volume in eyes with angioid streaks measured by swept source optical coherence tomography. Am J Ophthalmol.

[9] Unterhuber A, Povazay B, Hermann B, Sattmann H, Chavez-Pirson A, Drexler W (2005). In vivo retinal optical coherence tomography at 1040 nm—enhanced penetration into the choroid. Opt Express.

[10] Agawa T, Miura M, Ikuno Y, Makita S, Fabritius T, Iwasaki T (2011). Choroidal thickness measurement in healthy Japanese subjects by three-dimensional high-penetration optical coherence tomography. Graefes Arch Clin Exp Ophthalmol.

[11] Mrejen S, Spaide RF (2013). Optical coherence tomography: imaging of the choroid and beyond. Surv Ophthalmol.

[12] Gliem M, Fimmers R, Müller PL, Brinkmann CK, Finger RP, Hendig D, Holz FG, Charbel Issa P (2014). Choroidal changes associated with Bruch membrane pathology in pseudoxanthoma elasticum. Am J Ophthalmol.

[13] Chan CY, Papakostas TD, Vavvas DG (2014). Choroidal changes associated with Bruch membrane pathology in pseudoxanthoma elasticum. Am J Ophthalmol.

[14] Gallego-Pinazo R, Dolz-Marco R, Gómez-Ulla F, Mrejen S, Freund KB (2014). Pachychoroid diseases of the macula. Med Hypothesis Discov Innov Ophthalmol.

